# Shk (a histidine kinase) positively regulates the virulence of *Ralstonia solanacearum* strain GMI1000

**DOI:** 10.3389/frmbi.2025.1605947

**Published:** 2025-09-11

**Authors:** Dexing Xue, Danyu Kong

**Affiliations:** Jiangxi Provincial Key Laboratory of Plant Germplasm Resources Innovation and Genetic Improvement, Lushan Botanical Garden, Chinese Academy of Sciences, Jiujiang, China

**Keywords:** *Ralstonia solanacearum*, quorum sensing, pathogenesis, Shk, PhcA

## Abstract

Quorum sensing (QS) serves as a regulatory system of virulence factors in the *Ralstonia solanacearum* species complex (RSSC). The two-component system PhcS–PhcQ recognizes QS signals, subsequently activating the transcriptional regulator PhcA and promoting the expression of QS-dependent virulence factors. In this study, we identified a sensor histidine kinase (Shk) in the *R. solanacearum* strain GMI1000 and uncovered its essential roles in PhcA-dependent virulence. To investigate the functions of Shk in QS-dependent virulence, we generated an *shk*-deletion mutant (Δ*shk*) and demonstrated that the deletion of *shk* leads to a lowered production of cellulase, biofilm, and exopolysaccharide. Moreover, the complementation of native *shk* in Δ*shk* cell restored the QS-dependent phenotypes. However, the swarming motility of Δ*shk* cells was significantly increased compared to the wild-type GMI1000 strain. The Δ*shk* mutant exhibited impaired colonization of *R. solanacearum* in the xylem vessels of tomato plants, resulting in attenuated pathogenicity of Δ*shk* to tomato plants. Consistent with the results of the virulence assay, the deletion of the *shk* gene of *R. solanacearum* led to the downregulation of the *phcA*, *epsB*, and *cbhA* genes *in planta*, while the expression of *fliC* was upregulated in the Δ*shk* mutant relative to the wild-type GMI1000 strain. Pull-down assays suggested that RSc0040 functions as a response regulator for the sensor Shk *in vivo* and *in vitro*. Collectively, Shk is implicated in the regulation of these QS-dependent virulent factors, thereby contributing to the virulence of *R. solanacearum* to tomato plants.

## Introduction

*Ralstonia solanacearum* is a causal agent of bacterial wilt disease that infects over 450 plant species, including both dicots and monocots (Mansfield et al., 2012; Kim et al., 2016). The soil-borne pathogen thrives in tropical, subtropical, and warm temperate regions worldwide, posing significant threats to agricultural productivity (Tsuzuki et al., 2023; Wang et al., 2023). *Ralstonia solanacearum* infects plant roots through wounds or cracks at infected sites and multiplies within the xylem vessels, thereby disrupting water conductance and causing plant wilting eventually (García et al., 2019; Planas-Marquès et al., 2020). Among the top 10 plant bacterial pathogens, *R. solanacearum* ranks second due to its scientific/economic importance (Mansfield et al., 2012). Virulent determinants of *R. solanacearum* depend on swarming/twitching motility, cell wall-degrading enzymes (CWDEs), dozens of type III effectors (T3Es), and exopolysaccharides (EPSs) (Lowe-Power et al., 2018; Landry et al., 2020; Paudel et al., 2020).

Quorum sensing (QS) controls the activities of the cell-to-cell community that is widely conserved in *R. solanacearum* (Yan et al., 2022a; Kai, 2023). At present, SolI/SolR, RasI/RasR, and PhcBSRQ QS systems have been identified in *R. solanacearum* (Flavier et al., 1997; Yan et al., 2022a). SolI synthesizes acyl-homoserine lactone (AHL) signals, which are recognized by the transcriptional regulator SolR, subsequently activating the expression of AHL-inducing genes in *R. solanacearum* GMI1000 (Khokhani et al., 2017; Kai, 2023). However, the SolI/SolR system does not contribute to the pathogenicity of *R. solanacearum* (Khokhani et al., 2017). The phenotype conversion (*phc*) system, comprising the QS sensory cascade PhcBSRQ and the global transcriptional regulator PhcA, regulates the modulation of virulent factors in R. solanacearum, such as EPS production, secretion of CWDEs, and biofilm formation (Kai et al., 2015; Kai, 2023; Takemura et al., 2023). Methyl 3-hydroxymyristate (3-OH MAME) and methyl 3-hydroxypalmitate (3-OH PAME), synthesized by PhcB, function as QS signals and are then recognized by the histidine kinase (HK) PhcS (Kai et al., 2015; Hikichi et al., 2017; Kai, 2023). Upon perception of 3-OH MAME, the PhcS transfers a phosphate group from a donor histidine to the response regulator PhcQ, thereby inducing the expression of PhcA-dependent virulence factors (Takemura et al., 2021; Li et al., 2022). A mutation in 230 histidine, which is the phosphorylation site of PhcS, causes the defect in QS-dependent virulence (Kai, 2023). The two-component system (TCS) PhcS/PhcR represses the production of secondary metabolites, e.g., ralstonins and ralfuranones, but does not activate the expression of QS-dependent genes (Takemura et al., 2021; Kai, 2023).

The LysR-type global regulator PhcA in the *R. solanacearum* species complex (RSSC) plays a vital role in QS-dependent pathogenicity (Brumbley et al., 1993; Kai, 2023). The PhcA controls various activities associated with the pathogenicity/virulence of the RSSC strains in response to cell density (Kai, 2023). When 3-OH MAME is at high concentrations, PhcA in *R. solanacearum* positively regulates the production of virulent factors such as biofilm, CWDEs, and EPS (Perrier et al., 2018; Kai, 2023; Huang et al., 2024). On the contrary, PhcA promotes swarming/twitching motility, polygalacturonase production, and secretion of T3Es at low cell density (Khokhani et al., 2017; Perrier et al., 2018). PhcK encodes an HK sensor that activates the transcriptional expression of *phcA*, independently of the PhcS/PhcR and PhcS/PhcQ systems (Senuma et al., 2020). RNA-seq transcriptome analysis revealed that the gene expression profile in Δ*phcK* cells is similar to that of Δ*phcA* (Senuma et al., 2020). Given that *phcK* is not conserved in RSSC strains, the expression of *phcA* is probably regulated by other HK sensors (Senuma et al., 2020).

In this study, we described that an HK sensor (RSp0220), which was designated as Shk, controls the pathogenicity of *R. solanacearum* GMI1000 to tomato plants. To elucidate the roles of Shk on QS-dependent virulence, we constructed the Δ*shk* mutant and Δ*shk*(*shk*) complementation strain, performed virulent assays, and analyzed the expression of QS-dependent genes in Δ*shk*, Δ*shk*(*shk*), and wild-type GMI1000 strains using reverse transcription-quantitative real-time PCR (RT-qPCR). In addition, we found that RSc0040 acts as a response regulator (RR) for Shk, based on liquid chromatography-tandem mass spectrometry (LC-MS/MS) and protein pull-down analysis. These findings indicated that Shk positively regulates the pathogenicity of *R. solanacearum* to tomato plants.

## Materials and methods

### Bacterial strains, plasmids, and growth conditions

The bacterial strains and plasmids used in this study are detailed in **Supplementary Table S1**. *Ralstonia solanacearum* strains were cultured in CTG medium (casein acid hydrolysate, 1 g/L; tryptone, 10 g/L; and glucose, 5 g/L) at 28°C. The CTG agar plate was supplemented with 0.005% 2,3,5-triphenyltetrazolium chloride (final concentration). The upstream and downstream flanking regions of the *shk* gene and the spectinomycin resistance gene sequence were amplified by PCR with the primer pairs shk-1F/shk-2R, shk-3F/shk-4R, and pHSE401spcF/pHSE401spcR, respectively, cloned into the pK18mobsacB vector using the Gibson Assembly^®^ Master Mix (NEB, Ipswich, MA, USA). The resultant plasmid was electroporated into *R. solanacearum*-competent cells, creating the Δ*shk* mutant via sacB counterselection (Chen et al., 2018; Yan et al., 2022b). Transformants were selected on 50-mg/L spectinomycin-containing CTG plates. The Δ*shk* mutant was verified by PCR and DNA sequencing. DNA fragment of native *shk* was amplified by PCR using the primer pairs shk-5F/shk-6R with *R. solanacearum* GMI1000 genomic DNA as the template. The resultant plasmid was electroporated into the Δ*shk*-competent cell to generate an *shk-*complemented Δ*shk*(*shk*) strain. Similarly, a DNA fragment of the *shk* gene, fused with a 6×His tag sequence, was amplified by PCR using the primer pairs shkHis-7F/shkHis-8R and subsequently cloned into the pK18mobsacB vector. The recombinant plasmid was transformed into the Δ*shk* mutant, yielding the *shk-His* strain. A DNA fragment of the shk gene fused with the Flag sequence was amplified by PCR using the primer pairs shk-FlagUPF/shk-FlagUPR and shk-FlagDownF/shk-FlagDownR, and then cloned into the pK18mobsacB vector, yielding the pK18mobsacB-*shk*-*Flag* vector. Furthermore, DNA fragments of the *RSc0291* and *RSc0040* genes fused with a 6×His sequence were amplified by PCR with the primer pairs RSc0291UPF/RSc0291UPR/RSc0291-CDSF-HIS/RSc0291-CDSR-HIS/RSc0291DownF/RSc0292DownR and RSc0040UPF/RSc0040UPR/RSc0040-CDSF-His/RSc0040-CDSR-His/RSc0040DownF/RSc0040DownR, cloned into the pK18mobsacB-*shk-Flag* vector, thereby yielding pK18mobsacB-*shk-Flag-RSc0291-6×His* and pK18mobsacB-*shk-Flag-RSc0040-6×His* vector, respectively. The resultant plasmids were transformed into the Δ*shk* mutant, ultimately yielding *shk-Flag/RSc0291-6×His* and *shk-Flag/RSc0040-6×His* strains.

### Pathogenicity assay

Four-week-old tomato plants (Moneymaker) were inoculated with Δ*shk*, Δ*shk*(*shk*), and wild-type GMI1000 strains (10^9^ CFU/mL) by soil soaking with 20 mL of bacterial suspension as previously described by Takemura et al. (2023). The inoculated plants were then maintained in an incubator at 28°C with a 14-h light and 10-h dark cycle. Each treatment consisted of 20 plants, and the experiment was repeated three times. The disease symptoms were monitored and recorded daily according to the following disease index scale: 0, no wilting; 1, 1%–25% wilting; 2, 26%–50% wilting; 3, 51%–75% wilting; 4, 76%–99% wilting; and 5, dead.

### RNA extraction and RT-qPCR

Tomato plants were sectioned into 2 cm pieces, placed in a 2-mL tube containing 1 mL of sterile water at 4°C, and vigorously shaken to release the bacteria. Total RNA was extracted from plant-deprived *R. solanacearum* or 1 mL cultures of *R. solanacearum* (OD_600_ = 1) using the Eastep™ Super Total RNA Extraction Kit (Promega, Shanghai, China). Complementary DNA (cDNA) synthesis was performed with the HiScript III RT SuperMix for quantitative PCR (Vazyme, Nanjing, China). RT-qPCR analyses were carried out with the ChamQ Universal SYBR qPCR master mix (Vazyme, Nanjing, China). The transcript level of *gdhA* was used as a reference control for RT-qPCR analysis. The relative expression values of the target genes were calculated using the threshold cycle (2^−ΔΔCT^) method, as described by Yan et al. (2022a). The primers used in RT-qPCR analysis are detailed in **Supplementary Table S2**. RT-qPCR experiments were performed in triplicate.

### Detection of the numbers of *Ralstonia solanacearum* in plants

Tomato roots or stems were harvested at 5 days after inoculation (DAI), weighed, cut into small pieces, and then placed in tubes containing 1 mL of sterile water (de Pedro-Jové et al., 2021). After shaking for 20 min, the suspensions were serially diluted and spread on CPG plates. The plates were incubated at 28°C for 48 h. Subsequently, the numbers of *R. solanacearum* cells were counted. Each treatment consisted of 10 plants. The experiments were repeated in triplicate.

### Phenotype assays

EPS quantification was conducted as described previously (Song et al., 2022). In brief, *R. solanacearum* was cultured in a sucrose-peptone liquid medium (peptone, 5 g/L; sucrose, 20 g/L; KH_2_PO_4_, 0.5 g/L; MgSO_4_, 0.5 g/L; pH 7.2). A 100-mL aliquot of the culture (OD_600_ = 3.0) was harvested and subjected to centrifugation at 12,000 rpm for 20 min. The collected supernatants were mixed with 4 volumes of ethanol and incubated at 4°C overnight. The precipitated EPSs were isolated via centrifugation, dried at 55°C overnight, and weighed. The independent experiments were repeated in triplicate.

Biofilm formation was performed in polystyrene tubes as described previously (Song et al., 2022). In brief, the bacterial suspensions were adjusted to an OD_600_ of 0.2 with sterile water. The bacteria were cultured 1:20 in CTG medium in 96-well polystyrene plates, statically incubated at 28°C for 48 h, and finally stained with 0.1% crystal violet. The stained samples were repeatedly rinsed with sterile water and dissolved in 95% ethanol. Biofilm formation was quantified by measuring absorbance at 570 nm (*A*_570_). The independent experiments were repeated in triplicate.

Swarming motility was assessed on 0.3% semisolid agar plates described previously (Song et al., 2022). In brief, bacterial suspensions were adjusted to an OD_600_ of 0.2 with sterile water. Aliquots (1 µL) of the suspensions were added at the center of the tryptone-solidified medium (tryptone 10 g/L and agar 3 g/L). The plates were incubated at 28°C for 48 h before the diameter of the swarming halos was measured. The independent experiments were repeated in triplicate.

Cellulase activity was determined as described previously (Song et al., 2022). In brief, bacterial suspensions were adjusted to an OD_600_ of 0.2 with sterile water. Aliquots (1 µL) of the suspensions were added to carboxymethylcellulose sodium (CMS) solid medium (CMS 1 g/L, Na_3_PO_4_ 3.8 g/L, agar 8 g/L, pH 7.0). The plates were incubated at 28°C for 48 h. The plates were stained with 0.5% Congo red for 30 min. The plates were rinsed three times with 1 M of NaCl. The diameter of the transparent circles was observed and measured. The independent experiments were repeated in triplicate.

### LC-MS/MS analysis and pull-down assays

*Ralstonia solanacearum shk-His* cells were harvested from CTG medium (OD_600_ = 1) and 4-week-old tomato plants. The bacterial cells were centrifuged and resuspended in a lysis buffer (50 mM of NaH_2_PO_4_, 300 mM of NaCl, pH 8.0, and 1 mM of phenylmethylsulfonyl fluoride). The suspension was sonicated, centrifuged, and sequentially added with Ni-NTA magnetic agarose beads. The beads were then washed with a wash buffer (50 mM of NaH_2_PO_4_, 300 mM of NaCl, 10 mM of imidazole, pH 8.0). The Shk-6×His complex was eluted with an elution buffer (50 mM of NaH_2_PO_4_, 300 mM of NaCl, 200 mM of imidazole, pH 8.0) at 4°C. Finally, the protein complex was analyzed by the LC-MS/MS system (Thermo Scientific, Shanghai, China).

For the modified pull-down assay *in vivo*, the fusion gene constructs were transformed into Δ*shk* cells via electroporation as described by Wise and Binns (2016). The transformed cells were cultured and lysed in 10 mL of protein extraction buffer (150 mM of NaCl, 1% Triton X-100, 50 mM of Tris, pH 7.5 and 1× protease inhibitor cocktail) and sonicated on ice. The samples were centrifuged at 12,000 rpm for 15 min at 4°C. The supernatant was added with 50 µL of Ni-NTA magnetic agarose beads and incubated on a shaker at 4°C overnight. After incubation, the beads were washed three times with a wash buffer (150 mM of NaCl, 50 mM of Tris, pH 7.5). The input and bead-bound proteins were mixed with 6× SDS loading buffer and boiled for 5 min. The proteins were separated on 12% SDS-PAGE gels and detected by Western blot analysis with anti-Flag or anti-His antibodies.

For protein pull-down assay *in vitro*, GST, Shk-ID-6×His (intracellular domain of Shk), and GST-RSc0040 fusion proteins expressed in *Escherichia coli* (DE3) were purified by affinity chromatography as described by Liu et al. (2017). Fifty micrograms of GST, GST-RSc0040, was incubated with 50 µg of Shk-6×His in PBS buffer and subsequently added with 50 µL of glutathione agarose beads at 4°C under agitation for 2 h. The beads were collected by centrifugation and washed five times with pre-cooled PBS buffer. After washing, input and bead-bound proteins were detected by Western blot analysis using appropriate antibodies (Abcam, Shanghai, China).

### Statistical analysis

Statistical analyses were conducted with Prism 9 software (GraphPad, Boston, MA, USA). Results were presented as the mean ± standard deviation (SD). Statistical significance was determined with a two-tailed *t*-test for comparison between two treatments. *P*-value was used to denote the statistical significance of the differences: * indicates *P* < 0.05, and ** indicates *P* < 0.01.

## Results

### Shk contributed to *Ralstonia solanacearum* pathogenicity

**Figure 1:**
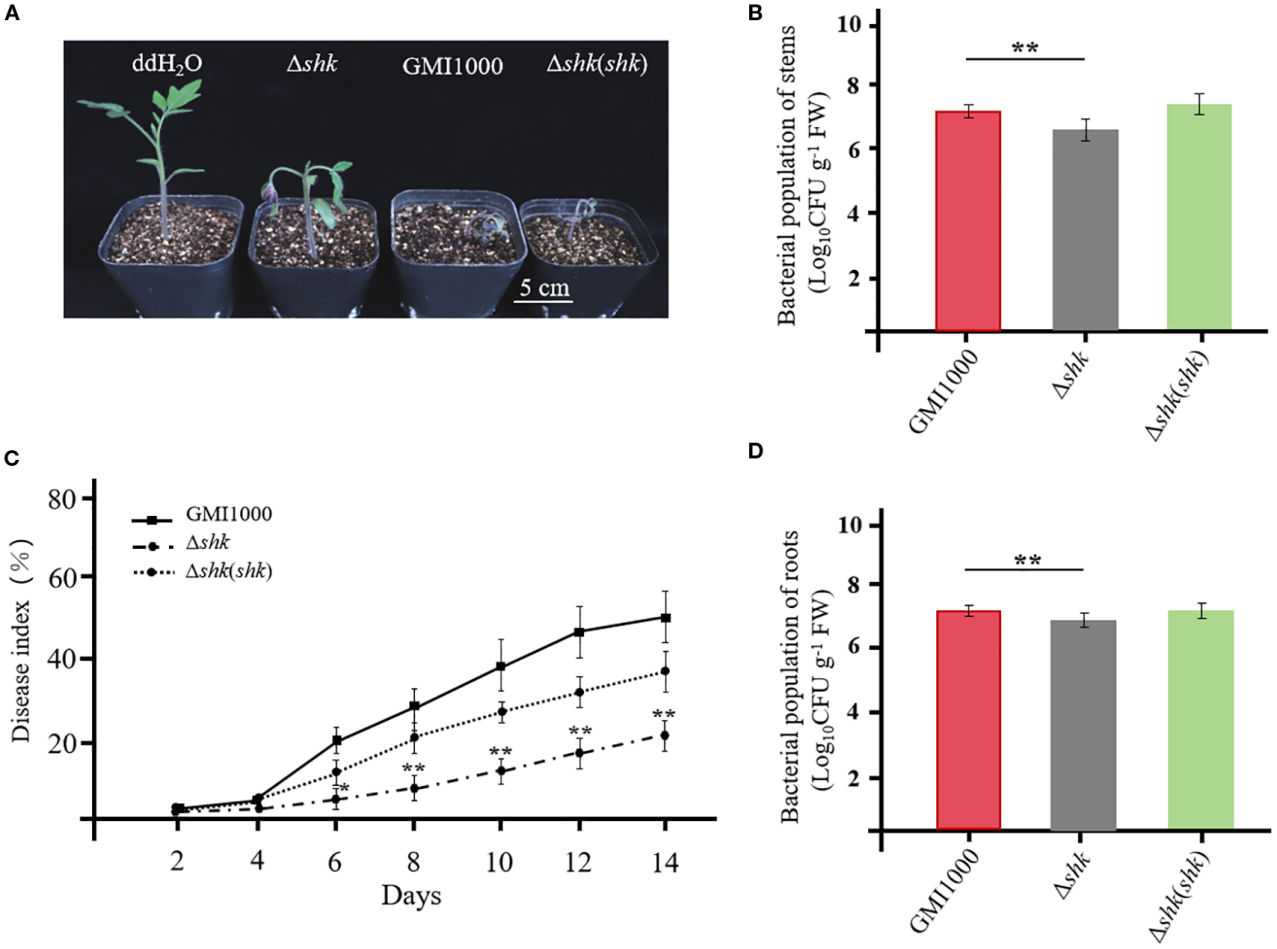
Shk pathogenicity of R. solanacearum to tomato plants. **(A)** Phenotypes of tomato plants at 5 days after inoculation of Ashk, Ashk(shk) and wild-type GMI1000 strains; **(C)** Disease index of tomato plants inoculated with Ashk, Ashk(shk) and wild-type GMI1000 strains. Populations of Ashk, Ashk(shk) and wild-type GMI1000 strains in the stems **(B)** and roots **(D)** of tomato plants at 5 DAI. Data are presented as the mean ± SD of nine trials. Asterisks indicate a significant difference from wild-type GMI1000 (*p < 0.05; **p < 0.01; t test).

We screened for virulent genes by analyzing dynamic transcriptional changes in *R. solanacearum* UY031 under different conditions and identified a virulent HK sensor RSUY_RS17350 (de Pedro-Jové et al., 2021). We found that RSUY_RS17350 mRNA expression in *R. solanacearum* UY031 cells within the early xylem and late xylem exhibited 2.6- and 2.8-fold upregulation compared to that in a rich medium. The RSUY_RS17350 was homologous to the HK sensor Shk in *R. solanacearum* GMI1000. To investigate the effects of Shk on *R. solanacearum* pathogenicity, 4-week-old tomato plants were inoculated with Δ*shk*, Δ*shk*(*shk*), and wild-type GMI1000 strains using the soil soak inoculation method. The tomato plants inoculated with *R. solanacearum* GMI1000 and complemented strain Δ*shk*(*shk*) exhibited apparent wilt symptoms at 5 DAI (**Figure 1A**). In contrast, tomato plants infected by the Δ*shk* mutant showed a significant reduction in wilt symptoms (**Figure 1A**). A disease index was employed to monitor the progression of bacterial wilt. As illustrated in **Figure 1C**, the disease indices of tomato plants inoculated with GMI1000 and Δ*shk*(*shk*) strains were 0.511 and 0.365 at 13 DAI. In contrast to both the GMI1000 and Δ*shk*(*shk*) strains, the Δ*shk* mutant exhibited a significant decrease in disease index, which was 0.218 at 13 DAI (**Figure 1C**). To further investigate the potential contribution of Shk to the colonization of *R. solanacearum in planta*, we quantified the bacterial colony-forming units (CFUs) in the stems and roots of tomato plants. The cell numbers of the Δ*shk*(*shk*) and GMI1000 strains in tomato stems were 7.06 × 10^7^ CFU/g and 1.77 × 10^8^ CFU/g at 5 DAI, respectively (**Figure 1B**), whereas the cell numbers of the Δ*shk* mutant in tomato stems were 2.31 × 10^7^ CFU/g at 5 DAI, which were significantly lower than those of the Δ*shk*(*shk*) and GMI1000 strains (**Figure 1B**). The CFUs of the Δ*shk*(*shk*) and GMI1000 strains in tomato roots were significantly increased in comparison to those of the Δ*shk* mutant at 5 DAI (**Figure 1D**). These findings suggested that Shk is crucial for the pathogenicity of *R. solanacearum*.

### Expression of virulent factors was modulated by Shk

**Figure 2:**
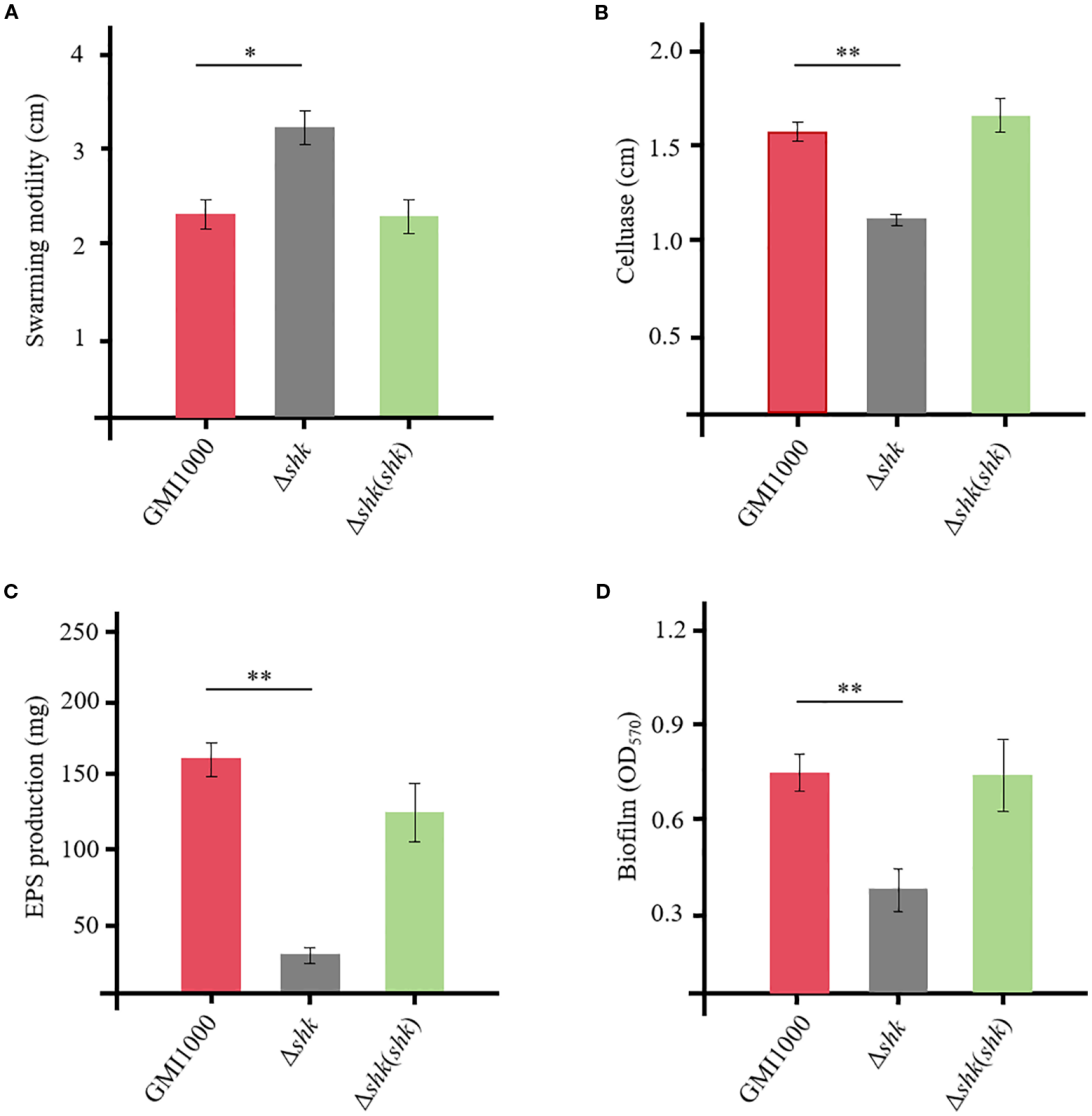
QS-dependent virulent factors of R. solanacearum GMI1000 and derivatives. **(A)** swimming motility; **(B)** cellulase production; **(C)** EPS production; **(D)** biofilm formation. Data are presented as the mean ± SD of three trials. Asterisks indicate a significant difference from wild-type GMI1000 (*p < 0.05; **p < 0.01; t test).

To assess whether Shk regulates the expression of virulence factors in *R. solanacearum*, we investigated its key biological functions, including swarming motility, cellulase activity, biofilm formation, and EPS production. The Δ*shk* mutant exhibited a significant increase in swarming motility compared to the wild-type GMI1000 strain (**Figure 2A**). Complementation of *shk* fully restored the phenotypic trait of the Δ*shk* mutant to wild-type GMI1000 levels (**Figure 2A**). The deletion of *shk* led to a marked decrease in cellulase activity (**Figure 2B**), EPS production (**Figure 2C**), and biofilm formation (**Figure 2D**) compared to the wild-type GMI1000 strain. In addition, these indices were restored to normal levels in the complemented strain Δ*shk*(*shk*). These findings revealed the important roles of Shk in regulating *R. solanacearum* virulence.

### Shk modulated the expression of virulence-related genes in *Ralstonia solanacearum*

**Figure 3:**
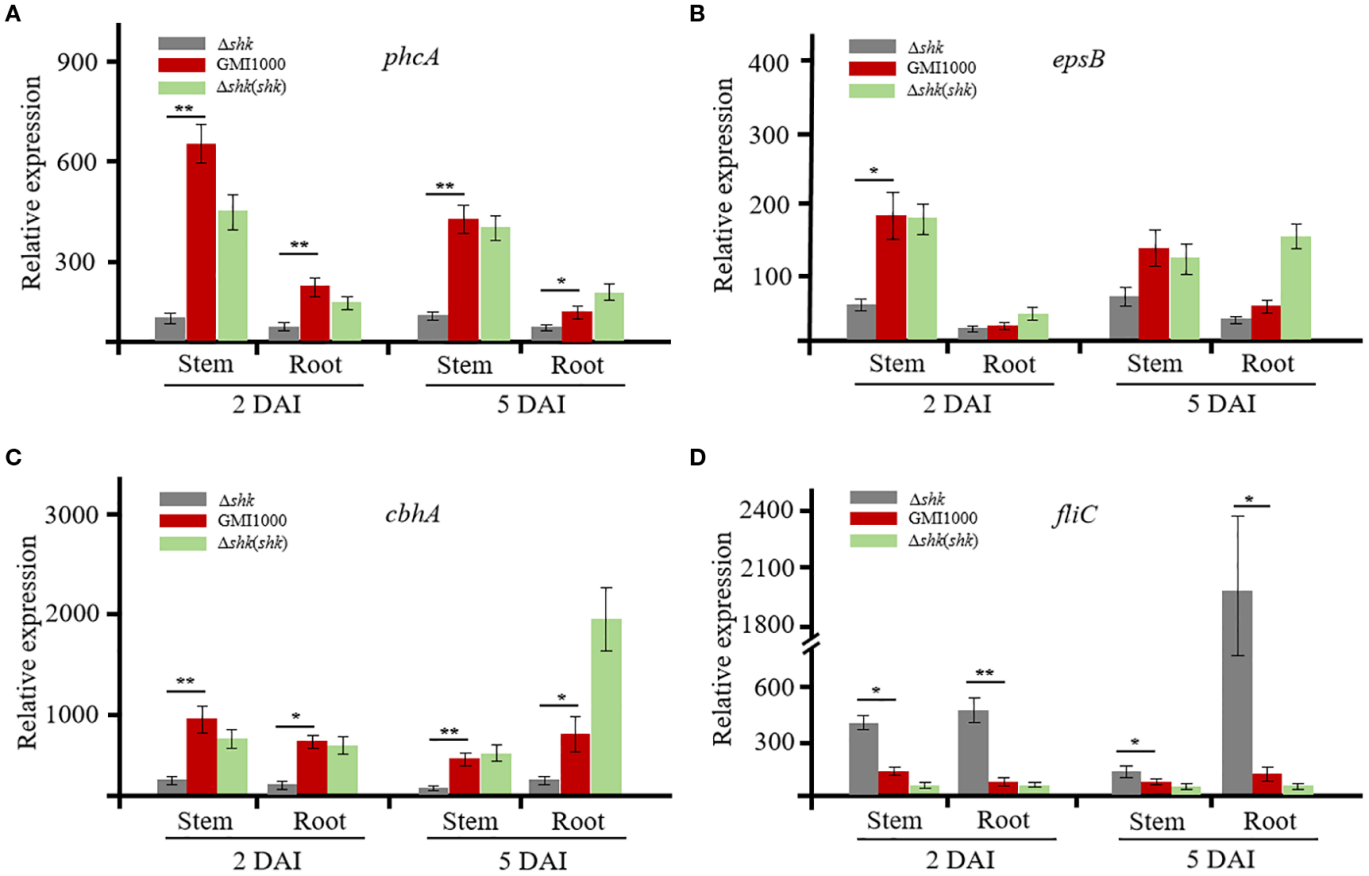
Expression of QS-dependent genes of R. solanacearum GMI1000 and derivatives in planta. **(A)** Transcript levels of phcA in Ashk, Ashk(shk) and wild-type GMI1000 strains; **(B)** Transcript levels of epsB in Ashk, Ashk(shk) and wild-type GMI1000 strains; **(C)** Transcript levels of cbhA in Ashk, Ashk(shk) and wild-type GMI1000 strains; **(D)** Transcript levels of fliC in Ashk, Ashk(shk) and wild-type GMI1000 strains. Gene transcript levels were normalized to that of gdhA. The experiments were performed with three biological replicates and two technical replicates. Data are presented as the mean ± SD of three trials. Asterisks indicate a significant difference from wild-type GMI1000 (*p < 0.05; **p < 0.01; t test).

To unveil the regulatory role of Shk on QS-dependent genes, we conducted RT-qPCR analysis to quantify the transcript levels of *phcA*, *epsB* (EPS I polysaccharide export protein), *cbhA* (1,4-β-cellobiosidase), and *fliC* (flagellin protein) in *R. solanacearum*, which were released from infected tomato plants. At 2 DAI, the *phcA* transcript level in wild-type GMI1000 cells was approximately 5.7-fold lower than that of Δ*shk* cells harvested from tomato stems (**Figure 3A**). The *phcA* expression in Δ*shk* cells from tomato stems at 5 DAI was significantly reduced relative to wild-type GMI1000 cells (**Figure 3A**). Notably, complementation of *shk* in the Δ*shk* mutant restored *phcA* expression to wild-type GMI100 levels. Similar expression patterns of the *phcA* were observed in tomato roots (**Figure 3A**). The transcript levels of *epsB* and *cbhA* in the Δ*shk* mutant derived from the roots at both 2 DAI and 5 DAI were significantly downregulated compared to the Δ*shk*(*shk*) and wild-type GMI1000 strains (**Figures 3B, C**). Likewise, *epsB* and *cbhA* were expressed at lower levels in Δ*shk* cells harvested from tomato stems at 2 DAI and 5 DAI than those in Δ*shk*(*shk*) and wild-type GMI1000 strains. Similar results of *epsB* and *cbhA* were observed in tomato roots (**Figures 3B, C**). However, the transcript levels of the *fliC* in the Δ*shk* mutant derived from the roots at both 2 DAI and 5 DAI were significantly higher than those in the Δ*shk*(*shk*) and wild-type GMI1000 strains (**Figure 3D**). Similar expression patterns of the *fliC* were detected in tomato stems (**Figure 3D**). These findings indicated that Shk positively modulates the expression of *phcA*, *epsB*, and *cbhA*, while it negatively regulates the expression of *fliC* of *R. solanacearum in planta*.

To determine whether the expression levels of *phcA*, *epsB*, *cbhA*, and *fliC* were regulated by Shk in CTG medium, we also used RT-qPCR to analyze the four QS-dependent genes. When the bacterial density reached an OD_600_ of 1, *R. solanacearum* cells were harvested for RT-qPCR analysis. As illustrated in **Supplementary Figures S1A, D**, the transcript levels of the *phcA* and *fliC* genes did not exhibit significant differences among the Δ*shk*, Δ*shk*(*shk*), and GMI1000 strains. However, the expression of *epsB* and *cbhA* in Δ*shk*(*shk*) cells was moderately upregulated compared to that of the Δ*shk* and GMI1000 strains (**Supplementary Figures S1B, C**). These findings suggested that the RNA expression patterns of the QS-dependent genes *phcA*, *epsB*, *cbhA*, and *fliC* of *R. solanacearum in planta* differ from those grown in CTG medium.

### Shk interacted with the response regulator RSc0040

The two-component system is one of the major signal transduction pathways in *R. solanacearum*, which consists of an HK and an RR. Extracellular signals are sensed by the HK, leading to the phosphorylation of the downstream RR and ultimately triggering the expression of virulence factors. Shk was predicted to possess seven transmembrane regions, a histidine phosphotransfer domain (DHp), and a histidine kinase-like ATPase domain (HATPase) according to the SMART (Simple Modular Architecture Research Tool) web service. To identify the Shk-interacting response regulator, we isolated the Shk-6×His complex from CTG medium and plant-derived *R. solanacearum* cells and further conducted LC-MS/MS analysis. As shown in **Supplementary Table S3**, four response regulators—PhcR, VsrC, RSc0291, and RSc0040—were identified in the Shk complex from the medium-derived *R. solanacearum* cells. To confirm these findings, we proceeded with LC-MS/MS analysis of the Shk-interacting complex in *R. solanacearum* cells released from tomato plants. The LC-MS/MS analysis revealed that the response regulators RSc0291 and RSc0040 were also co-precipitated with Shk-6×His in plant-derived *R. solanacearum* cells (**Supplementary Table S3**).

**Figure 4:**
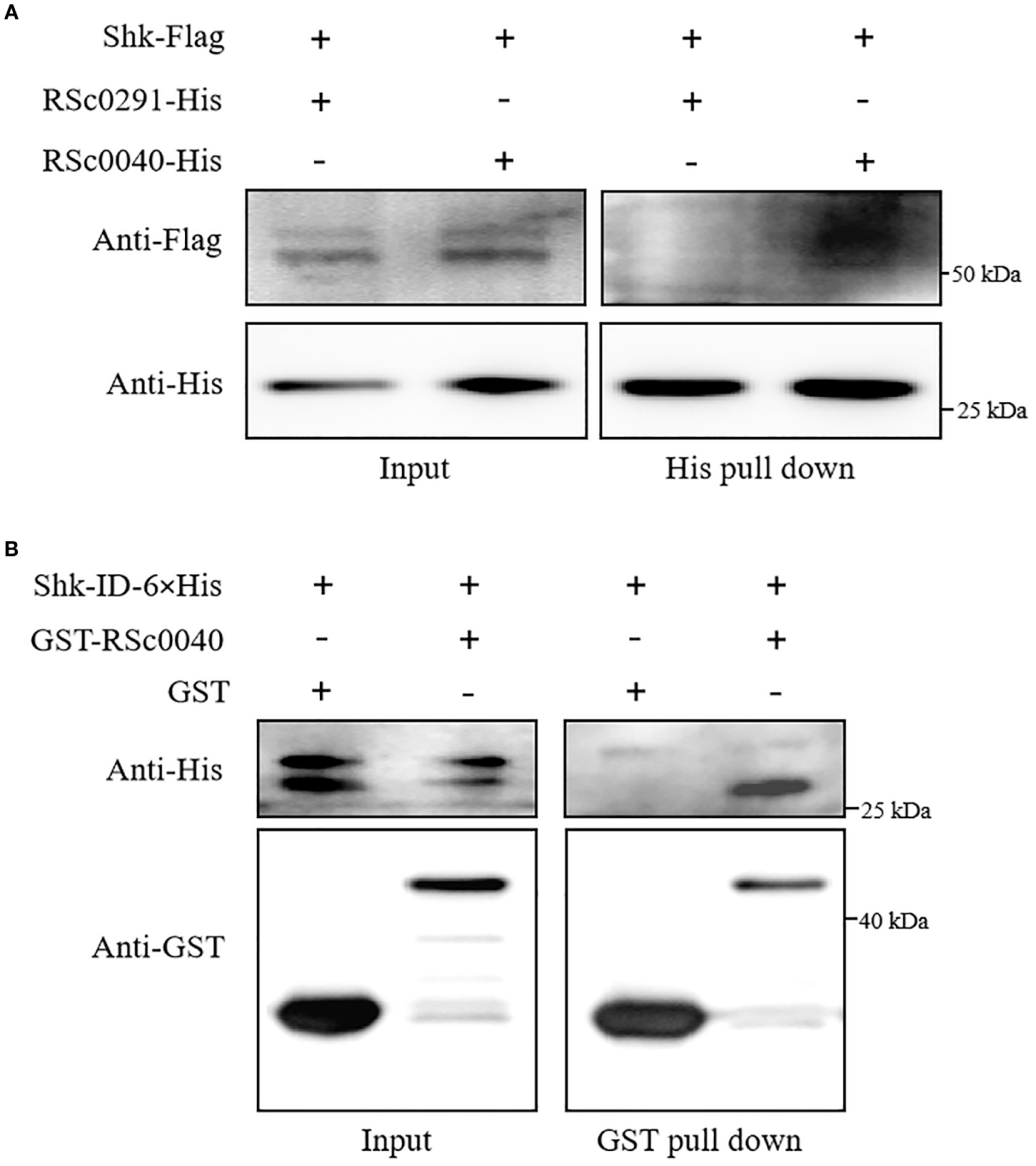
Shk interaction with response regulator RSc0040. **(A)** Pull-down assay of the interaction between Shk-Flag and RSC0040-6×His or RSC0291-6×His in vivo. The recombinant Shk-Flag/RSc0040-6×His and Shk-Flag/RSC0291-6×His expressed in R. solanacearum Ashk cells were subjected to pull-down analysis. The recombinant proteins was detected by anti-Flag and anti-His immunoblotting; **(B)** GST pull-down assay of the interaction between Shk-ID-6×His and GST-RSC0040 in vitro. Shk- ID-6×His, GST-RSC0040 and GST were expressed in E. coli. Purified proteins were co-incubated for 2 h and pulled down with glutathione agarose beads. Western blot analysis was performed to detect the input and bead-bound proteins using appropriate antibodies.

To further substantiate the interaction between Shk and these response regulators, we performed a modified pull-down assay to assess the interactions between Shk and RSc0291 or RSc0040. For this purpose, constructs of *shk-Flag/RSc0291-6×His* and *shk-Flag/RSc0040-6×His* were respectively cloned into the vector pK18mobsacB and subsequently introduced into Δ*shk* cells via electroporation. As shown in **Figure 4A**, the co-precipitation of Shk-Flag with RSc0040-6×His using Ni-NTA beads was successfully detected with anti-Flag antibody *in vivo*. However, no interaction was observed between Shk-Flag and RSc0291-6×His (**Figure 4A**). These results indicated that the sensor Shk forms a direct association with the response regulator RSc0040 *in vivo*.

To evaluate whether the sensor Shk interacts with the RSc0040, pull-down assays were carried out *in vitro*. Shk-ID-6×His, GST-RSc0040, and GST were expressed in *E. coli* and purified with affinity chromatography. In order to test whether the used pull-down assay is appropriate, we analyzed the Shk-ID-6×His interaction with GST alone. The result showed that Shk-ID-6×His did not associate with GST. The results of the pull-down assays with the recombinant proteins showed that Shk-ID-6×His was pulled down by GST-RSc0040 (**Figure 4B**), indicating that the Shk was directly associated with the response regulator RSc0040 *in vitro*.

## Discussion

Three QS systems, PhcBSRQ, SolI/R, and RasI/R, have been identified in RSSC strains (Yan et al., 2022a; Kai, 2023). The PhcBSRQ QS system is recognized for its key roles in regulating the chemotaxis system, swarming/twitching motility, EPS production, CWDE production, type III secretion system (T3SS), and biofilm formation (Corral et al., 2020; Inoue et al., 2023; Kai, 2023). The global virulence regulator PhcA is vital to the Phc QS system, acting as a molecular switch to modulate the production of virulence factors (Kai, 2023).

In this study, a novel TCS sensor Shk was identified and characterized in the *R. solanacearum* strain GMI1000. Phenotypic analyses revealed that Shk is crucial for regulating various biological activities associated with bacterial virulence, including swarming motility (**Figure 2A**), cellulase production (**Figure 2B**), EPS production (**Figure 2C**), and biofilm formation (**Figure 2D**). Consistent with the biochemical findings, virulence assays demonstrated that deletion of the *shk* gene in the *R. solanacearum* strain GMI1000 significantly attenuated its pathogenicity (**Figures 1A, C**). Root and stem colonization assays indicated that the CFUs of the Δ*shk* mutant in stems and roots were significantly lower than those of the Δ*shk*(*shk*) and GMI1000 strains (**Figures 1B, D**), suggesting that Shk is essential for *R. solanacearum* colonization in tomato plants.

In agreement with the findings of the virulence assay, RT-qPCR analysis in this study showed that the deletion of the *shk* gene in the *R. solanacearum* strain GMI1000 resulted in downregulation of the transcript *cbhA* (**Figure 3C**). Exopolysaccharides produced by *R. solanacearum* accumulate within the xylem vessels, obstructing water flow and ultimately leading to severe wilting symptoms (Shi et al., 2023). The *epsABCDEF* cluster and multiple activators (XpsR, VsrD, and VsrC) are required for EPS production (Hikichi et al., 2017; Kai, 2023). In the Δ*shk* mutant, the transcript level of *epsB*, which is essential for EPS I biosynthesis, was significantly reduced compared to the Δ*shk*(*shk*) and GMI1000 strains (**Figure 3B**). The finding was in strong agreement with the virulence assay results, which demonstrated that the deletion of the *shk* gene diminishes EPS production. Notably, the complement of the *shk* gene in the Δ*shk* mutant resulted in increased transcript levels of *epsB* and *cbhA in vitro* (**Supplementary Figures S1B, C**). Biofilm formation, a critical determinant of *R. solanacearum* virulence, was significantly reduced in the Δ*shk* mutant relative to the Δ*shk*(*shk*) and GMI1000 strains (**Figure 2D**), suggesting that Shk contributes to biofilm production. The deletion of *shk* does not affect the expression levels of the *phcA* gene *in vitro*, suggesting that Shk regulates the expression of the transcriptional regulator *phcA* via plant-derived signals (**Supplementary Figure S1A**). Together, RT-qPCR assays indicated that the deletion of *shk* results in significant reductions in the expression levels of the QS-dependent genes *phcA*, *epsB*, and *cbhA* of *R. solanacearum in planta*.

Swarming motility, driven by rotating flagella, is essential for *R. solanacearum* pathogenicity (Corral et al., 2020). The RT-qPCR results demonstrated that Shk negatively regulates the *fliC* expression (**Figure 3D**), which encodes the flagellar filament structural protein in *R. solanacearum*. This finding aligned with the observed impact of Shk on bacterial swarming motility in the virulence assay. Meanwhile, the mRNA expression pattern of the *fliC* gene was similar to that of *phcA*, suggesting that Shk does not modulate *fliC* expression *in vitro* (**Supplementary Figure S1D**). Analysis of the virulent phenotype *in vitro* revealed significant differences between the Δ*shk* and wild-type GMI1000 strains (**Figure 2**). However, the expression levels of the QS-dependent genes *phcA*, *epsB*, *cbhA*, and *fliC* in Δ*shk* and wild-type GMI1000 cells cultured in CTG medium did not change remarkably (**Supplementary Figure S1**). It is hypothesized that the inconsistency is probably attributed to environmental conditions and the timing of *R. solanacearum* cell culture.

Four RRs, PhcR, VsrC, RSc0291, and RSc0040, were pulled down with Shk, suggesting that Shk possibly establishes direct or indirect associations with these response regulators in CTG medium-derived *R. solanacearum* cells (**Supplementary Table S3**). The second LC-MS/MS results further showed that the two response regulators RSc0291 and RSc0040 form complexes with Shk of *R. solanacearum in planta* (**Supplementary Table S3**). Pull-down assays *in vivo* and *in vitro* confirmed a direct interaction between the Shk and RSc0040, suggesting that RSc0040 is a response regulator for the sensor Shk (**Figure 4**).

In conclusion, this study identified Shk as an HK sensor in the *R. solanacearum* strain GMI1000. Shk contributes to the pathogenicity of *R. solanacearum* to tomato plants and positively regulates its essential abilities related to EPS synthesis, cellulase production, and biofilm formation, while it negatively regulates swarming motility. These findings provide novel insights into the role of Shk in regulating the virulence of *R. solanacearum* in a PhcA-dependent manner and present a foundational basis for the control of bacterial wilt diseases.

## Data Availability

The datasets presented in this study can be found in online repositories. The names of the repository/repositories and accession number(s) can be found in the article/**Supplementary Material**.
