# Supplementary figures and images for: Shk (a histidine kinase) positively regulates the virulence of Ralstonia solanacearum strain GMI1000
Source: Front Microbiomes. 2025 Sep 11;4:1605947. doi: 10.3389/frmbi.2025.1605947 (PMC12993560; doi:10.3389/frmbi.2025.1605947)

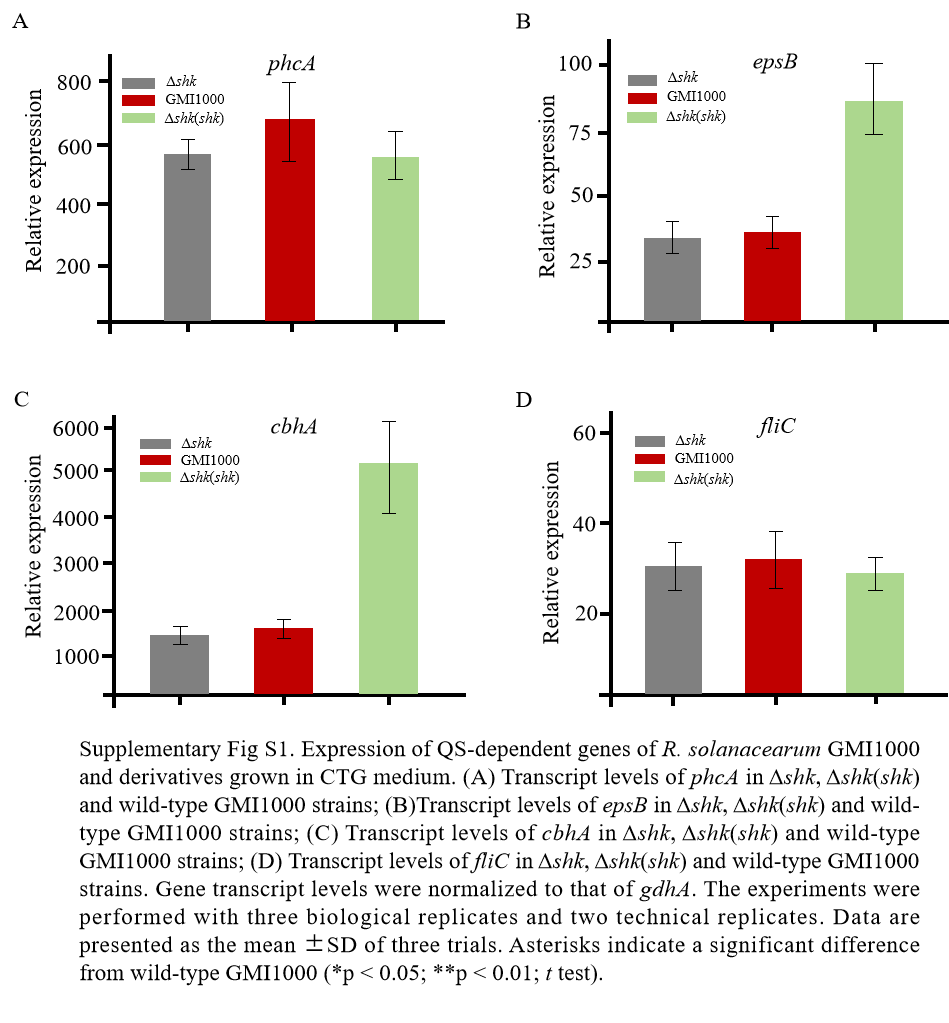

Supplement: Supplementary file 1 [file Image1.tif]

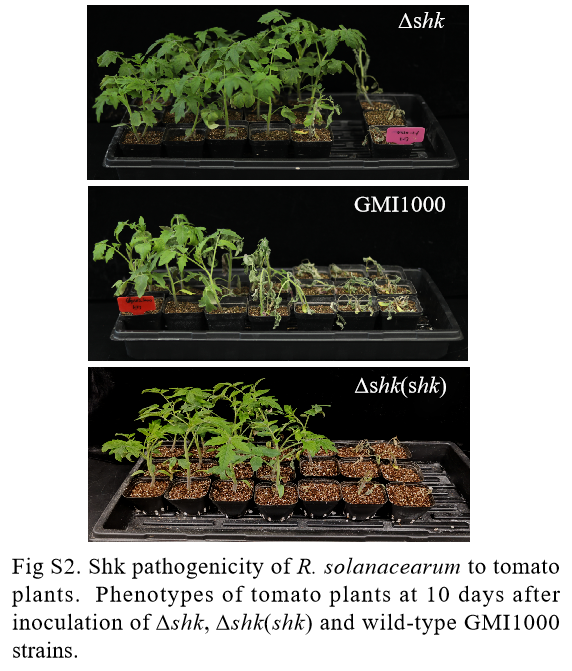

Supplement: Supplementary file 2 [file Image2.tif]
